# International recruitment of mental health nurses to the national health service: a challenge for the UK

**DOI:** 10.1186/s12912-022-01128-1

**Published:** 2022-12-12

**Authors:** Peter Phiri, Sana Sajid, Ardic Baykoca, Suchith Shetty, Daisy Mudoni, Shanaya Rathod, Gayathri Delanerolle

**Affiliations:** 1 Research & Innovation Department, Southern Health NHS Foundation Trust, Southampton, UK; 2 Psychology Department, Faculty of Environmental and Life Sciences, University of Southampton, Southampton, UK; 3 Nuffield Department of Primary Care Health Sciences, University of Oxford, Oxford, UK

**Keywords:** Mental health nurses, International nurses, International recruitment, Nursing shortages

## Abstract

The UK’s National Health Service (NHS) has been dealing with a shortage in the nursing workforce for the past few decades. With the ongoing COVID-19 pandemic and post-Brexit effects, it is important now more than ever to concentrate on recruiting new staff and retaining current staff in the National Health Service. The increasing demand for mental health services stresses the importance of prioritising recruitment of mental health nurses. One of the main strategies being implemented to combat this shortage is the recruitment of internationally trained mental health nurses. Whilst this is a favourable solution, the multiple challenges this proposal brings makes it hard for the National Health Service to practically implement this to increase staff numbers. In this discursive position paper, we consider the difficulties the National Health Service is currently facing in terms of recruiting mental health nurses and then discuss the importance of and need for international recruitment including the strategies that are currently being implemented. The challenges and obstacles associated with this proposed resolution will also be addressed.

## Introduction

The prevalence of mental health disorders and its burden on the NHS is increasing rapidly with time. Within England, 1 in 6 people experience a common mental health problem in the space of a week and as of May 2022, Alzheimer’s and Dementia are the leading causes of death in England (11.0%) [1, 2]. In most cities in England, between 9.4% and 11.7% of inhabitants stated that they experience some type of common mental health problem, with trends showing a continuous increase in mental health issues across England [1]. In March 2020, prevalence of moderate or severe depression across Great Britain was 10%, which increased to 19% in June 2020 and further increased to 21% in March 2021 [3]. Similarly, there was a 5.8% increase in children aged 6–16 who were thought to have a probable mental health illness [4]. There was also an increase in the percentage of teenagers aged between 17 and 19 with a probable mental health disorder, from 10.1% in 2017 to 17.4% in 2021. These statistics show a persistent increase in mental health problems across Great Britain which is further reflected in the increased engagement in mental health services. NHS mental health services were accessed by around 2 million adults and 0.8 million children in 2020/21 and the increasing rates of mental health problems will only inflate the amount of engagement with mental health services [1].

The growing demand for such services is not being met by the healthcare system within the UK, largely due to inadequate staff numbers. At present, there are over 125,000 individuals employed within NHS England that work within the mental health services sector, the largest group being registered mental health nurses [5]. In the last decade however, there has been a 5% reduction in the number of occupied nursing positions, with Mental Health Trusts in the UK holding a vacancy rate of 16% [5, 6]. The Royal College of Nursing conducted a survey amongst 9,577 of their registered nurses, health care support workers, students, and nursing associates to understand and analyse employment experiences within health care settings [7]. They found that 74.1% of respondents regularly worked beyond their contracted hours and 17.4% mentioned this occurring every working day/shift [7]. Most of these extra hours are unpaid and the overloaded pressure has meant that 37% of staff are unable to take their full annual leave entitlement [7]. Over three quarters of staff reported working at least once on a day where they should have taken sick leave within the previous 12 months and of these individuals, 66.8% reported illness due to stress and 37.9% reported illness due to mental health issues [7]. Moreover, voluntary resignation was reported to have increased by 88% whilst nurses felt unable to achieve a ‘work-life balance’. [7].

The Conservative Party’s election manifesto for the 2019 General Election pledged to recruit 50,000 more nurses for the NHS in England by 2024/25 [8]. The manifesto did not specify if the number of nurses would suffice both the clinical and research needs of a growing population in the UK, leaving room for uncertainty [8]. It seems the general trend of recruitment is going in the opposite direction, with fewer admissions and retentions to mental health nursing programs [7]. Individuals who do train on these programs find that the bulk of education is solely focused on the physical health aspects of nursing, with little consideration for how mental health services would operate and be delivered within the role [9]. This could mean that mental health nurses enter the NHS with less training compared to their physical health nurse colleagues [9]. Aside from this, student nurses are required to complete a large requirement of placement hours, and although the government are aiming to increase the uptake of students into the NHS, the quality of such placements is questionable [8, 9].

Training more staff, retaining the existing nursing workforce, and increasing international recruitment are strategies to be used to achieve this target, which included financial incentives for struggling disciplines such as mental health, including support with childcare costs [10].

There are currently 38,888 mental health nurses employed by NHS England in March 2022 [8], and the target number to reach by 2023/24 is 56,467 [11]. This means that, of the 50,000 nurses that the Conservative Party pledged to hire, over 16,000 must be mental health nurses. The nursing crisis was highlighted in the recent Health Foundation report noting that the government target of 50,000 will be insufficient to meet the current nursing workforce demand, which is furthered by the pandemic [12]. It is questionable that mental health services are not receiving their fair share of NHS funding considering that 23% of total health services burden is due to mental health problems but only 14.8% of the health budget was allocated to this sector in 2021/22 [13].

In this discursive paper, we aim to address one of the strategies pledged by the Conservative Party due to reasons for brevity—increasing international recruitment. We will discuss rationale behind this proposal, possible shortcomings of this solutions and other available routes which may help to target the shortage of mental health nurses within the NHS.

### International recruitment within the NHS

International recruitment provides the NHS with a stream of staff, including nurses, coming to England to live and work. This strategy remains an important part of the plan to increase workforce numbers within NHS organisations, as stated in the NHS People Plan [14]. The NHS Long Term Plan outlines the aims and ambitions of the NHS across the next decade, highlighting international recruitment as a priority for the workforce [15]. For the NHS to continue to grow, it is vital that health services integrate the recruitment of international nursing staff in their plans for the upcoming years [14].

Many international healthcare professionals elect to travel to the UK for various benefits. When hired by the NHS, international nurses receive a guaranteed 3-year visa and sponsorship from the organisation, with contracts often getting renewed and staff continuing in their positions for many years [16]. Their visa also supports applications for dependents, so staff can have their partners and children join them in the UK [16]. Annually, many nurses leave their roles due to the disparity between high workload and low pay, and this is a huge problem in developing countries [17]. An enticing factor to working within the UK is the competitive salary offered which, when compared to salary rates from developing countries, is very generous [16, 17].

**Fig. 1:**
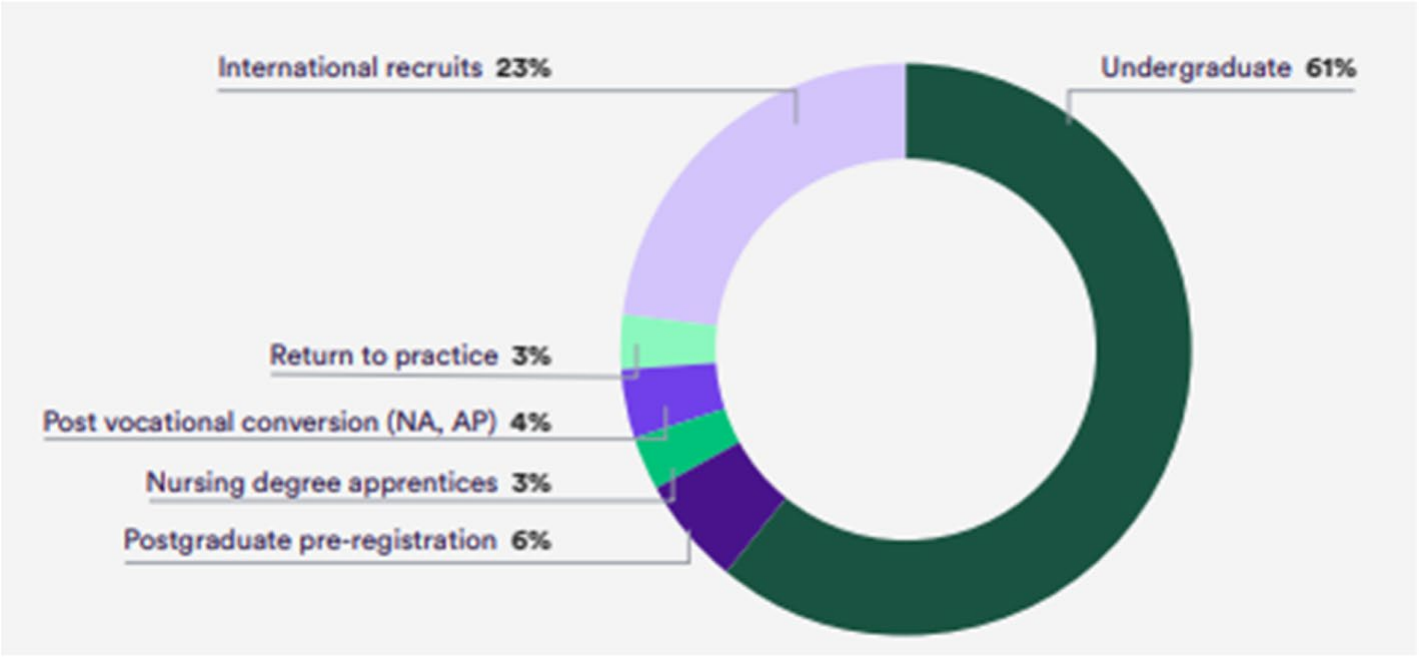
Annual supply of registered nurses in England. (Source: Health Foundation, Nuffield Trust [18]). Legend: Graphic depiction of the percentage of career backgrounds of registered nurses in England

There are various routes which can be taken to achieve the goals outlined in the NHS People Plan such as improved nurse retention, re-recruitment of previous NHS staff, and increased number of domestically trained nurses [14]. International recruitment offers a much faster route to nurses initiating practice as training nurses domestically can take at least three years or even longer. In order for this to be a viable proposal, there needs to be an increase of ethical international recruitment and adherence to its policies [14]. At present, 23% of NHS nurses working with hospital or community settings are overseas national and are already playing an invaluable role within the UK healthcare system and as shown in Fig. 1, these numbers are set to increase further [18].

The total upfront costs of recruiting an international nurse range from around £10,000 to £12,000 which, in comparison, is much cheaper to the £26,000 spent on training a UK-based nurse for an undergraduate training post or even £140,000 spent by Trusts that hire apprentice nurses [15]. Around 15% of NHS staff are not British nationals with the UK recruiting maximum numbers of foreign nurses (~ 10,000/year), followed by the USA and Germany yet the nursing remains to be on the Shortage Occupation List for the 6^th^ year running [19, 20]. An evaluation concluded that while international recruitment addresses immediate staff shortages, it is not sustainable as a long-term solution on a large scale [21]. Ideally, there shouldn’t be an overreliance on international recruitment, but it should be encouraged for healthy cultural exchange and given its current situation, that is an ambitious goal for the NHS to achieve.

### Current strategies for international recruitment

There are various strategies that the NHS uses to attract international nurses. Firstly, entry barriers have been eased for international staff. Through the Health and Care Visa scheme launched by the UK in August 2020, it is quicker, affordable, and easier for registered international healthcare staff to come to work in the UK. Furthermore, the Skilled Worker visa allows individuals trained sectors of high UK demand to move over to the UK for work purposes and emigrate their spouse or children in addition as dependents [20]. The Nursing International Recruitment Programme initiative aids and supports NHS organisations to increase and develop their international recruitment plan. This program supports NHS Trusts with access to international market, and with innovative and collaborative approach to recruitment. For instance, the program launched NHS Pastoral Care Quality Award in March 2022 to promote high-quality pastoral care to international nurses [22]. NHS England and NHS Improvement is incentivising Trusts to build local hubs with a lead-recruiter and system-level models to promote international recruitment. These hubs are also meant to provide support to new starters who travel to work from overseas. Other small-scale initiatives include the Refugee Nurse Support Pilot Programme which supports refugees who are qualified as nurses in their home country to resume their nursing careers in the NHS [23]. Likewise, the Global Learners Programme offers support packages for practicing nurses from abroad to move to the UK to work in the NHS [24].

### Difficulties with international recruitment

According to the International Council of Nurses (ICN) report published on 24^th^ January 2022, an estimated 13 million more nurses will be needed worldwide in the next decade [25]. In the World Mental Health Report, released be the World Health Organisation, this global shortage of mental health nurses is preventing the adequate care of individuals all over the globe [26]. Essentially, there is worldwide demand for nurses and NHS is in direct competition with other countries in this aspect. In this section we look at different factors that affect international recruitment and how the NHS fares with its competition.

### Lack of international mental health nurses

**Fig. 2:**
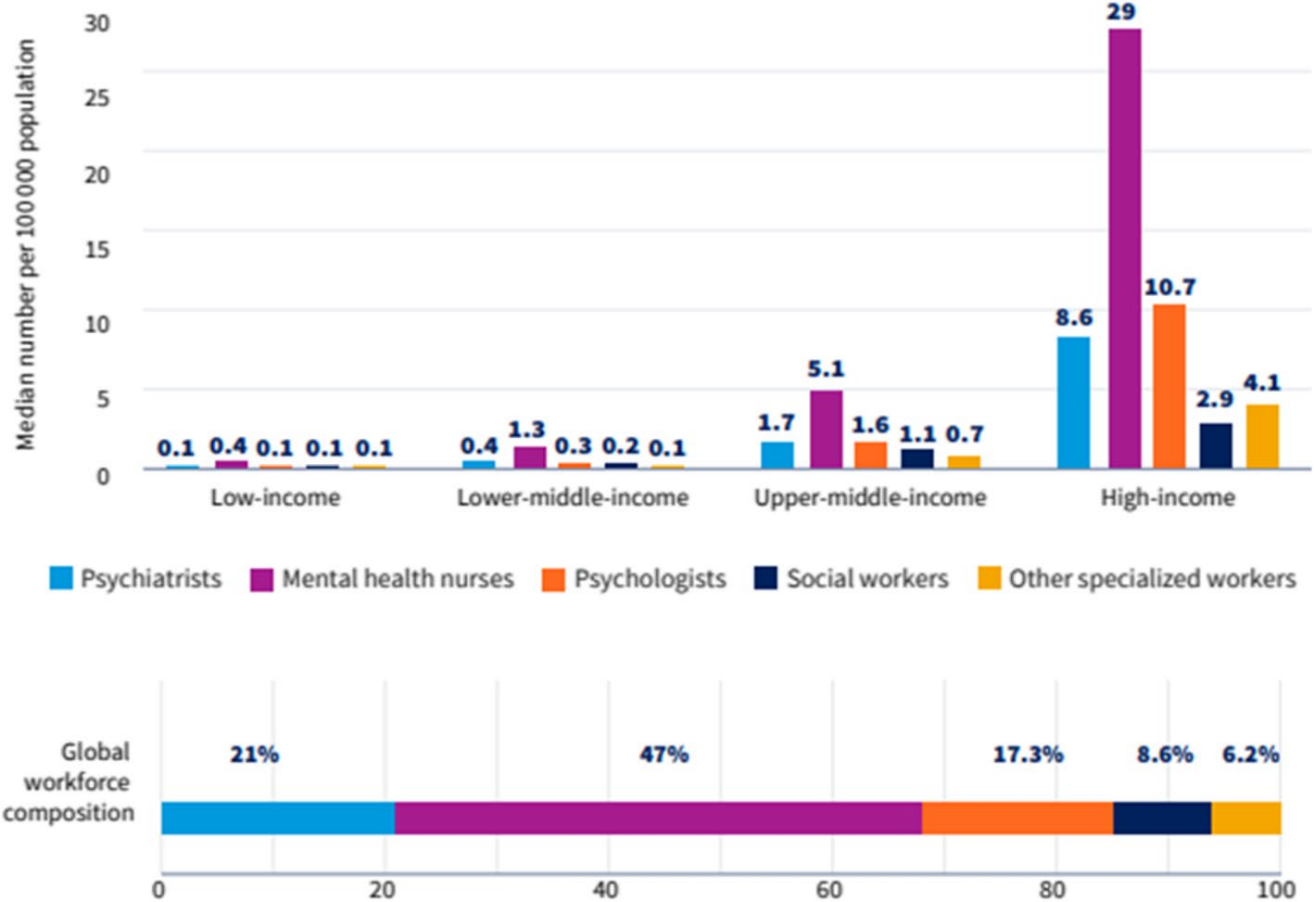
specialised mental health workforce across country groups. (Source: World Health Organisation [27]). Legend: The graph above represents the proportions of various mental health professionals across different countries, split into income brackets. Each bar number represent the number of professionals per 100,000 members of the population. The graph at the bottom displays the global percentages of various mental health professionals. Permissions: Fig. 2 was sourced from the World Health Organization (WHO) [27]. Their copyright statement reads as follows: *Permission from WHO is not required for the use of WHO materials issued under the Creative Commons Attribution-Non-Commercial-ShareAlike 3.0 Intergovernmental Organization (CC-BY-NC-SA 3.0 IGO) license. For further information, please see: Copyright (who.int)*

One of the issues with trying to recruit overseas mental health nurses is that they are in short supply globally with many countries struggling to hire various professionals to deal with the mental health demand [27]. In developing countries, the ratio of mental health professionals to the population is 1:100,000 compared to the ratio of 1:60 within developed countries [27, 28]. Figure 2 shows the lack of international mental health nurses which contributes to the difficulties in hiring such professionals [27].

### Training requirements

If mental health nurses wish to migrate to work within the NHS, there is an extensive eligibility criterion which must be met in order for the nurses to be approved for work. Alongside the scarcity of international mental health nurses, the selective list of requirements again blocks off roads for opportunity for many. Currently, the UK requirements for overseas mental health nurses are as follows [28]:

- Approved by UK Immigration (Visa successfully issued)
- Qualification equivalent to a UK Level 1 Nurse (e.g., completed a three-year programme working towards a nursing qualification and a degree/diploma)
- Test of Competence (Computer-Based Test & Objective Structured Clinical Examination)
- English Language requirements
- Health Requirements
- Character requirements
- ID and DBS Check

All foreign applicants need to clear the above requirements in order to be approved to work as a nurse within the NHS. Report of pass rates for tests taken between July 2021 and September 2021 shows that the Computer-Based Test has a pass rate of ~ 90% and Objective Structured Clinical Examination pass rate is ~ 75% [29]. Most developed countries have a similar application and approval process for recruiting overseas nurses. While nurses who have qualified in developed countries can easily meet the criteria of other developed countries, it is those from developing foreign countries that may struggle in the face of the rigid criteria. The training and qualifications provided in developing countries are not recognised as suitable equivalents by developed countries; nurses would have to undergo extra training requirements, exams, and tests in order to be accepted to work overseas. In addition to the standardised Tests of Competence, Australia assesses eligibility based on a relevant nursing degree, the quality of recognizing authority, and the equivalent academic level of the degree in Australian standards as prescribed by the Nursing and Midwifery Board of Australia [30]. In the United States, foreign-trained nurses with accredited credentials need to clear a Qualification Exam offered by Commission on Graduates of Foreign Nursing Schools (CGFNS) to meet immigration requirements and then clear a Licensure Exam unique to the US State where they intend to work [31]. In comparison, Canada requires foreign-trained nurses need to complete a bridging course to be eligible for nursing registration [32].

### Ethical concerns of international recruitment

**Fig. 3:**
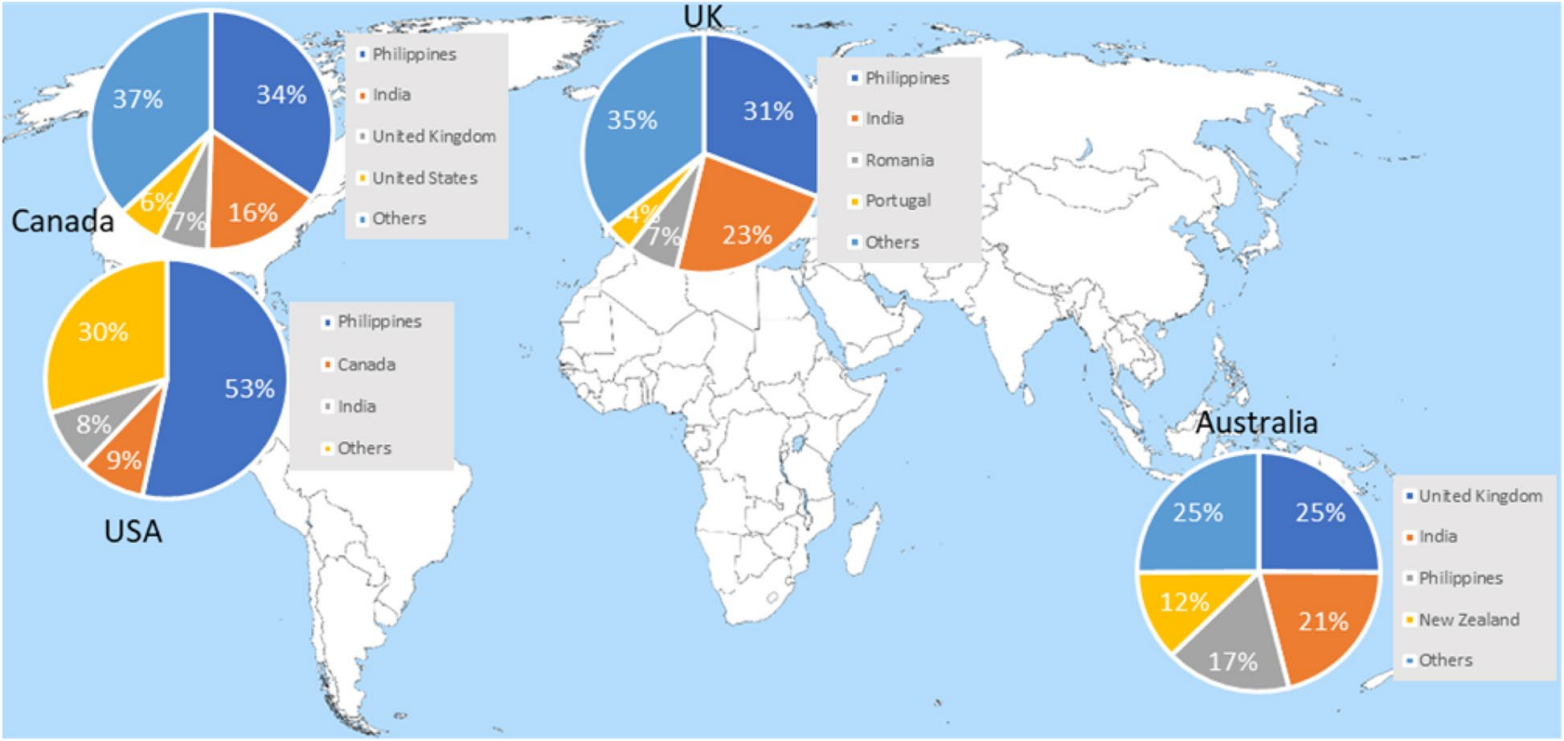
Distribution of foreign-trained nurses in developed countries by country of origin. (Source: OECD [36]). Legend: This map depicts the percentage of foreign-trained nurses in various developed countries across the globe. Foreign nurses are split by the country from which they trained. Permissions: This figure was created by the authors using data from the OCED website [36]. Hence, no further permission statement from OCED is required

As it stands, there are ethical concerns with the process of international recruitment, in particular with the notion of ‘poaching’ [33]. ‘Poaching’ is when an employer, such as the NHS, actively approaches individuals who are currently employed within another agency, with the aim to recruit them. This most commonly occurs with healthcare professionals from Low- and Middle-Income Countries (LMICs) [33, 34]. Mass migration is the reality over the last decade or so, where economic and political instability has caused many families, including healthcare professionals to seek a home within the developed part of the world. Thus, the attraction of a job in the world’s second-largest employer, the NHS can be alluring. This has caused debate around sustainable development and goals across LMICs. For example, out of 102,000 non-EEA nurses in the UK, ~ 38,000 are from the Philippines and ~ 32,000 are from India, together contributing to 69.6% of overseas nursing professionals [35]. The Philippines and India are notable LMICs with a growing population and deficits within their own healthcare systems. Most nurses around the globe have migrated to a developed country in order to continue their career (see Fig. 3) [36] In essence, the strategic objectives of developed countries to increase their healthcare workforce through various avenues could impact Sustainable Development Goals that the World Health Organisation (WHO) promotes [37]. Universal healthcare and equitable access to all clinical areas is a basic human right and is important legislatively. To this part, the UK is committed to ensure ethical recruitment practices by adhering to the UK Code of Practice for international recruitment, wherein active recruiting from some of the developing countries based upon WHO’s Workforce Support and Safeguard List, 2020 is restricted [37].

The UK has adopted an innovative digital recruitment process but in order to utilise this service to effective and ethically to employ international recruits, some adjustments could be made. The Workforce Support and Safeguard List must be always adhered to so the UK could promote to staff from countries where there is no or reduced restriction. They could also make full use of the capacity of recruits they are given in restricted countries.

### Disparities in wages

**Fig. 4:**
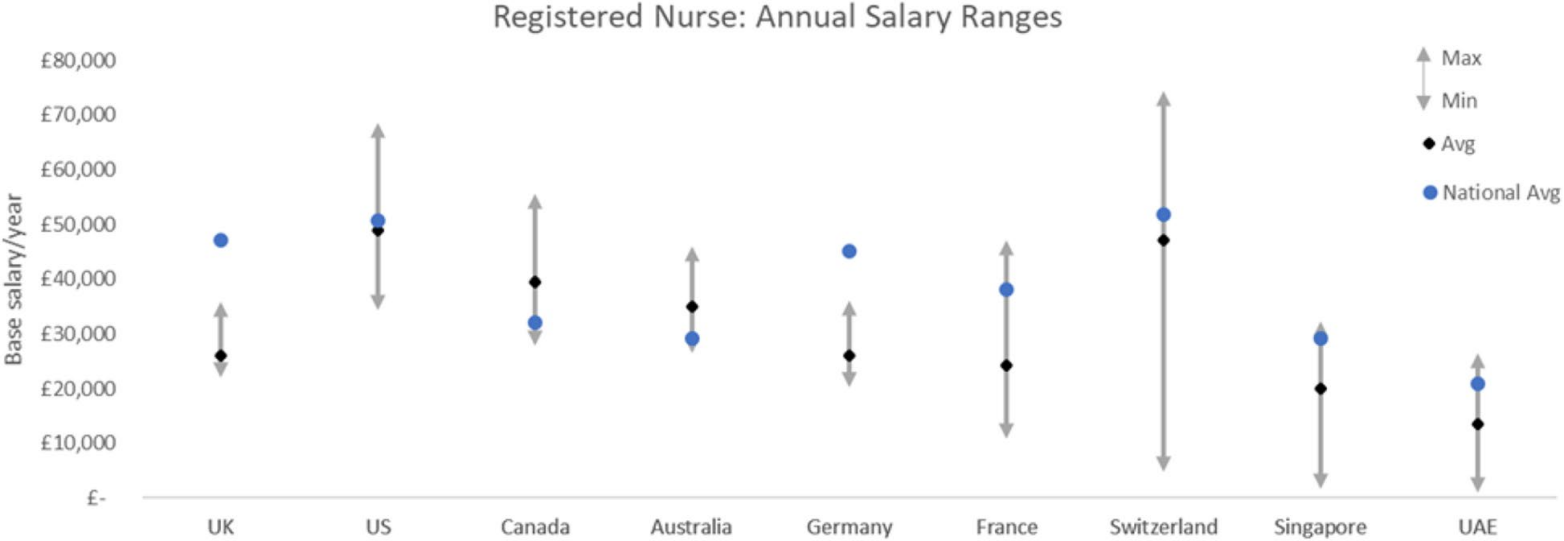
Annual salary ranges for Registered Nurse across various countries. (Source: OECD [36]. This graph shows the minimum and maximum salary ranges for registered nurses in various developed countries. The overall average salary has been indicated alongside the generic national salary average. All currencies have been converted to British Sterling (£)

Moving abroad for overseas recruitment warrants the consideration of one’s changes to salary and wages. From the perspective of nurses migrating to work in another country, it is thus important to consider what the differences in wages will be. The wages of a nurse vary across private and public sectors and largely depend on professional development and years of experience and recruiting country (see Fig. 4) [36].

For a ballpark comparison, the average annual wage of a nurse in the UK is around £35,000 with mental health nurses earning an average salary of £34,500, with an entry-level salary of £30,500 which can go up to £50,500 with experience [38]. In contrast, in the US mental health nurses earn an average of £46,500, with an entry-level salary of £39,000 and a late-career salary of £52,000 [39]. Few other countries with higher average pay for nurses are Luxemburg (£68,000), Denmark (£65,000), Canada (£56,000), Australia (£52,000), and Switzerland (£48,500) [40]. As displayed above in Fig. 4, the salary of nurses in the UK is considerably lower across levels of experience and the type of nursing. Therefore, it would be wise to offer more benefits to overseas mental health nurses to increase the number of incoming applications. This will be crucial in appearing more attractive to international recruits.

### Barriers surrounding cultural accommodations

Cultural differences can make it extremely challenging for overseas mental health nurses who have essentially spent all their lives living and working within a certain cultural environment. A study by Alexis (2013) reported that nurses experience intense culture shock when they began working within the NHS and found that they had to learn new techniques and approaches to working with UK-patients as it appeared to be very different to their former healthcare system [41]. There may be differences in terms of medical equipment, standard operating procedures, and approbation routines [42]. The NHS requires an abundance of paperwork to the extent to which staff feel they spend more time doing paperwork rather than providing physical care to patients; many overseas staff can find overwhelming [42]. In many cultures, caring for the elderly was a role adopted by the family and occurred within their natural community setting. In the UK however, it is community nurses who often provide this care to the elderly [43]. This may mean that nurses are not always experienced at working with elderly patient in a clinical setting. Due to cultural differences, it is quite challenging for overseas nurses to adapt to the values and norms that underpin the British health-care system. Whilst it is important to accommodate for these differences, in terms of practicality, this may be quite difficult. The mental health nursing within NHS is complex, operating across various services and settings with both international and home-based staff and patient groups.

For internationally recruited nurses, driving long distances to work or using the car to drive to various community settings may seem like a daunting task and a perceived obstacle to working in the NHS [44]. Although some steps have been made to accommodate for nurses of various cultural backgrounds, there is still a long way to go. Several Trusts have implemented new Internationally Educations Nurses positions within inpatient units to reduce the need to drive to various locations for work [44]. Where inpatient positions are not readily available, schemes are in place which enrol international nurses to community settings which can easily be reached via buses [44]. Although not an ideal solution, it shows the inclination to begin to accommodate for international nurses who may not be adapted to the cultural norms. A more specific example of where support has been implemented to assist international nurses with the cultural differences has been implemented by Norfolk Community Health and Care NHS Trust [45]. They compiled a 30-page guide, introducing Norfolk, with details of transport options and amenities of interest, as well as guidance on routes into driving within the UK and support for physical, spiritual, and mental wellbeing [46]. Such resources and the availability of extra training for professional development would assist international nurses in adjusting to working within the NHS. By providing with more information and exposure to the mental health settings, international recruits could have a more supported induction into their roles.

### Working conditions

Working conditions have a direct impact on productivity, performance, mental health, job satisfaction, and morale. Evidently, reputation of a safe and supportive work environment is likely to motivate a migrant nurse in search of a better life.

Internationally recruited nurses often felt marginalised and underappreciated by their UK-trained counterparts [47]. Overseas minority ethnic nurses often felt they were given the more challenging or demanding tasks leaving them feeling helpless and hesitant to raise their concerns and perceptions with senior staff [47]. Reports from 2019 suggest that stress, absenteeism, turnover, and intention to quit are at alarming levels amongst nurses in England [48]. Nurses are at a higher risk of harassment and bullying, particularly among staff with the minority ethnic background. To quote another alarming statistic, the risk of suicide was 23% higher in female nurses compared to national average over the period 2011 to 2015 [49]. It is useful to note that these conditions within the NHS are not unique to this organisation. A literature review found that 46.4% of top 50 articles on Workplace Violence included nurses in their sampling population [50]. In addition, a cross sectional survey of nurses in 12 European countries found that 1 in 5 nurses were dissatisfied with their jobs in most countries [51]. This highlights the need for urgent action from global organisations and governments as well as national bodies, and health and social care leaders to ensure that workplaces support, value and respect the healthcare staff and the services they provide for us.

## Outlook

At present, there is a global shortage of nurses, especially those specialised in providing mental health services and the UK is also one of those countries struggling to increase their staff numbers. Whilst some studies have shown that short-staffed hospital shifts display larger resilience to and occurrence of incidents, the National Institute for Health and Care Excellence have concluded that there is a need for high-quality studies illustrating the relationship between numbers of nursing staff and outcomes [52–54]. Overall, evidence shows higher staff numbers lead to beneficial outcomes, without no indication of the threshold number of staff required [54, 55]. Although the relationship between nurse staffing numbers and patient safety including incident rates is well recognised, nursing staffing level tools in acute medical settings are not applicable to mental health nursing staffing levels, as the uniqueness of mental health nursing adds complexity to tools to aid staffing decisions [56–60]. There remains paucity of evidence on tools suited to psychiatric intensive care units (PICU) and learning disabilities [56–60].

International recruitment is used by various organisations and services to attract overseas individuals to migrate in the pursuit of a new job and the NHS is included in this. Many NHS staff are employed through international recruitment schemes, and this is also being implemented for foreign-trained mental health nurses. Recruiting international nurses is a cheaper option for the UK who have recently made it easier for overseas nurses to migrate over however higher salary prospects may mean candidates turn to other countries offering better incentives. There are now global protocols in place which restricts developed countries from hiring high proportions of overseas staff from developing countries, narrowing the route for international employment. When recruitment of overseas mental health nurses is successful, the NHS have ensured they have some form of induction programmes or support packages in line for new staff to ensure a smooth and welcoming transition. It is important to note that there is still a long way to go in terms of accommodating for the needs of international staff and recognising and understanding cultural differences is the first step needed. By implementing policies and training procedures to aid the transition from healthcare in their home country and healthcare within the NHS, international staff may feel better equipped and understood within their new workplace. All evidence and research reviewed above provides strategic implications to policymakers, recruiting organisations, and of course to mental health nurses (both domestic and international) about the complex proposal that is international recruitment.

## Data Availability

Data sharing is not applicable to this article as no datasets were generated or analysed during the current study.

## Abbreviations

NHS: National Health Service
HEE: Health Education England
FTE: Full-Time Equivalents
RN: Registered Nurse
LMIC: Low-Middle-Income Countries
HIC: High-Income Countries
OECD: Organisation for Economic Co-operation and Development
WHO: World Health Organisation
